# Generation of nanobodies against the F protein of respiratory syncytial virus and establishment of an indirect immunofluorescence assay

**DOI:** 10.1128/spectrum.02784-24

**Published:** 2025-03-25

**Authors:** Qianqian Wang, Dongxiang Hong, Entao Li, Zekai Cheng, Jiachen Zhang, Tengchuan Jin, Sandra Chiu

**Affiliations:** 1 Department of Laboratory Medicine, The First Affiliated Hospital of USTC, Division of Life Sciences and Medicine, University of Science and Technology of China 612146 , Hefei, Anhui, China; 2 Division of Life Sciences and Medicine, University of Science and Technology of China 612146 , Hefei, Anhui, China; 3 Key Laboratory of Anhui Province for Emerging and Reemerging Infectious Diseases, Hefei, Anhui, China; University of Manitoba, Winnipeg, Manitoba, Canada

**Keywords:** respiratory syncytial virus, nanobody, immunofluorescence

## Abstract

Respiratory syncytial virus (RSV) infection is a leading cause of acute respiratory infections and death in children and older adults. Currently, there is a lack of effective vaccines and antibody treatments on the market, and the clinical manifestations of RSV are indistinguishable from those of acute respiratory infections caused by other etiologies. Therefore, the prevention and treatment of RSV require not only effective vaccines but also simple, fast, cost-effective, and accurate detection and diagnostic methods. Given the small molecular weight, excellent antigen-binding specificity, and affinity of nanobodies, we successfully obtained a panel of nanobodies by expressing the RSV F protein, immunizing camel, and establishing a phage display library. Among them, F-E2 has been shown to bind specifically to the RSV F protein and can be used for Western blot, enzyme-linked immunosorbent assay, flow cytometry, immunohistochemistry, and immunofluorescence assays. Moreover, an RSV immunofluorescence detection method based on F-E2 has been established and proved to be highly specific, sensitive, cost-effective, and fast, with great application potential.

**IMPORTANCE:**

A respiratory syncytial virus (RSV) detection nanobody, F-E2, was successfully screened by constructing a dromedary camel immune library. F-E2 binds to RSV F with ng affinities and can be used for Western blot, enzyme-linked immunosorbent assay, flow cytometry, immunohistochemistry, and immunofluorescence assays. Importantly, a high-throughput RSV immunofluorescence detection method based on F-E2 has been established and proved to be highly specific, sensitive, cost-effective, and fast, with great application potential.

## INTRODUCTION

Respiratory syncytial virus (RSV) is one of the most common pathogens that causes lower respiratory tract diseases in infants, the elderly, and immunodeficient individuals worldwide (1, 2). In 2019 alone, there were an estimated 330 million episodes of RSV-related acute lower respiratory tract infection worldwide, resulting in 3.6 million hospitalizations and nearly 101,400 deaths in children younger than 5 years in the hospital (3). RSV, which seems to be very rare, is the number one cause of hospitalizations for respiratory infections in infants and young children, and in developed countries, RSV is the number one cause of all hospitalizations in children under 5 years of age (4). In addition, among the frail elderly, the annual infection rate is approximately 5%–10%, of which the incidence of severe illness requiring hospitalization is about 14.5%, and the fatality rate is 1.6% (5, 6), similar to the incidence of influenza, which results in a heavy medical cost and treatment and prevention burden on global public health.

RSV titer detection is one of the research bases for developing related vaccines and antibodies. At present, there are diverse methods for the diagnosis of RSV infection, including classical viral plaque assay, tissue culture infective dose 50% (TCID_50_), real-time PCR assay (RT-qPCR), immunofluorescence (IF) assay, etc. (7, 8). The plaque assay and TCID_50_ method are time-consuming, and RSV needs to be cultured for about a week; after producing a large enough visible plaque, the virus titer can be calculated after fixation and crystal violet staining (9–11). However, in practice, the plaque method is not stable, the formation of plaque is small, the boundary of plaque is not clear, and the requirements for cell types and culture media are strict. More importantly, errors in plaque recognition and manual counting often lead to poor repeatability of the experiment. The RT-qPCR assay has high sensitivity, strong specificity, and a short detection cycle. However, for RSV titer detection, viral RNA samples need to be extracted, and the operational process is complicated, which can easily lead to RNA degradation and contamination, resulting in false-positive results. The indirect immunofluorescence method uses a specific antibody to react with the antigen in the virus and a fluorescently labeled secondary antibody to bind to the specific antibody. It observes the fluorescence signal and performs quantification by analyzing the fluorescence intensity. This method is fast, sensitive, and specific, with good experimental repeatability. However, considering the high cost of antibodies when there are more samples, this method still needs to be further optimized and improved. Given the limitations of RSV diagnostic methods, it is difficult to promote them in clinical examinations. Therefore, it is urgent to establish a simple, fast, cost-effective, and accurate diagnosis and detection method.

Studies have found that *Camelidae* and cartilaginous fish, such as sharks, have natural heavy-chain antibodies, with one of the variable heavy chain domains identified as VHH, also termed nanobodies (12, 13). Compared with traditional antibodies, nanobodies have many advantages, such as being the smallest natural antigen-binding domain, having a molecular weight of about 15 kDa, which is 1/10 that of the traditional antibodies. Their small size allows them to bind to narrow epitopes that traditional antibodies are usually unable to reach (14). Therefore, nanobodies have better specificity and affinity than traditional antibodies. In addition, nanobodies have the advantages of good stability, high expression, low production cost, and easy engineering transformation (15). At present, a number of nanobodies and their engineered antibodies are in the clinical trial stage and may be used in the future for the diagnosis and treatment of diseases, such as tumors, autoimmune diseases, and viral infections (16–18).

Here, we focused on the development of a new generation of RSV detection antibodies and identified a nanobody F-E2 from a large phage display library based on the heavy chain variable domain (VHH) of camel antibodies. F-E2 specifically binds to the F-glycoprotein on the RSV surface. Moreover, F-E2 can be used in Western blot, enzyme-linked immunosorbent assay (ELISA), flow cytometry, immunohistochemistry (IHC), and IF-related experiments. On the basis of F-E2, we established a simple, rapid, and cost-effective detection method to further improve the specificity and accuracy, which is necessary to improve the prevention and control of RSV and provides a nice tool for research.

## MATERIALS AND METHODS

### Cells and viruses

Vero cells (BDBIO; C5168) were maintained at 37°C and 5% CO_2_ in Dulbecco’s modified Eagle’s medium (DMEM; VivaCell; C3103-0500) supplemented with 10% fetal bovine serum (FBS; VivaCell; BL201A) and 1% penicillin-streptomycin (Biosharp; BL505A). RSV A2 and B strains were kindly provided by Dr. Rui Gong (Wuhan Institute of Virology, Chinese Academy of Sciences). A clinically isolated HRSV A strain (GZ08-18, GenBank accession No. KP119747) was kindly provided by Dr. Ke Zhang (The Key and Characteristic Laboratory of Modern Pathogen Biology, Guizhou Medical University, Guiyang, China). Vero cells were infected with the RSV A2 and harvested at 5–7 days post-infection. Cells were lysed by rapid freezing and thawing three times. Cell debris was removed by centrifugation at 3,000 rpm for 10 min. Virus titers were quantified via indirect immunofluorescence assay, and virus stocks were stored at −80°C until use.

### Construction, expression, and purification of RSV (strain A2) F proteins

The RSV (strain A2) F protein (post F) gene sequence was kindly provided by Dr. Rui Gong (Wuhan Institute of Virology, Chinese Academy of Sciences). The coding sequence for RSV F was cloned into the mammalian expression vector PTT5, and PTT5-post F recombinant plasmid was obtained. Proteins were produced in HEK 293F cells by transient transfection. Briefly, the target plasmids were transfected into HEK 293F cells by using polyethylenimine (MACKLIN, P924174-1g) according to the manufacturer’s instructions, after which the cells were cultivated for 4 days at 37°C and 120 rpm in 5% CO_2_. The cell culture supernatant was collected by centrifugation at 5,000 rpm for 10 min at 4°C and subsequently filtered through 0.22 µm sterile syringe filters (Biosharp; BS-PES25-22-S). Nanobody was purified on a microprotein purification system (Union-Biotech [Shanghai] Co., Ltd.; UEV 25D) with a 5 mL NI-NTA (Bioworks) affinity column (binding buffer: 20 mM Tris, 500 mM NaCl, and 16 mM imidazole, pH 8.0; elution buffer: 20 mM Tris, 500 mM NaCl, and 400 mM imidazole, pH 8.0) and a HiTrap Protein A (Cytiva; 17040301) column (binding buffer: PBS, pH 7.4; elution buffer: 0.1 M ethanoic acid). Subsequently, the eluent corresponding to the absorption peak was collected, diluted with 2× SDS loading buffer ([TANON; 180-8210D] with fresh 10% β-mercaptoethanol), and denatured at 100°C for 10 min. SDS-PAGE was used to identify the target protein bands and their purity. Finally, the protein was concentrated using an Amicon Ultra Centrifugal filter (Merck KGaA), centrifuged at 4°C and 3,000 rpm, and replaced in PBS solution. The concentration was measured by NanoDrop One (Thermo Fisher Scientific), subpackaged, and stored at −80°C permanently.

### Panning and identification of nanobodies specific for post F

The large dromedary camel VHH phage libraries were constructed as previously described (19). Then, 100 µL of 0.1 mg/mL post F antigen was coated on a microplate (Beijing Yunpeptide Biotechnology; 100096H) and washed twice using PBS. Next, the plate was blocked with 5% skim milk in PBS. A total of 1 × 10^11^ pfu phage library was added per well for the first round of panning. The volume was 100 µL, and it was incubated for 1 h at room temperature (RT) with shaking. The wells were washed 30 times to clear the unbound phages with PBS supplemented with 0.1% Tween-20. The bound phages were collected by digestion with 100 µL of 0.5 mg/mL trypsin for 1 h at RT. The 100 µL positive phages were added to 2 mL *Escherichia coli* TG1 cells for infection and amplification. The selection process for the second round was similar to the first, with minor differences. The concentration of antigen was decreased by 10-fold, and the amount of input phage was 1 × 10^9^. Finally, 96 individuals from two rounds of panning were identified by monoclonal phage ELISA according to a previous description (19). The candidate positive clones were further sequenced for analysis.

### Western blot

The infected Vero cells were collected by rapidly freezing and thawing three times. The cell pellet was fixed and resuspended. Then, the sample was diluted with 2× SDS loading buffer (TANON; 180-8210D) with fresh 10% β-mercaptoethanol and denatured at 100°C for 10 min. The protein sample was separated by 10% SDS-PAGE and transferred to PVDF membrane (Millipore, IPVH00010, 0.45 µm). The membrane was blocked with 5% skim milk in PBST for 1 h and incubated with primary antibody F-E2 or secondary antibody (HRP-conjugated Affinipure Goat Anti-Human IgG (H + L); Proteintech; SA00001-17) for 1 h at room temperature under gentle shaking. The protein bands were detected by a super-sensitive ECL chemiluminescence solution (P&Q Science Technology, Shanghai, China).

### Enzyme-linked immunosorbent assay

ELISA has been widely accepted as a method for characterizing antibody binding activity. Briefly, 96-well plates were coated with 1 µg/mL RSV-specific antigen RSV A2 F (post F) overnight at 4°C. The following day, the plates were washed three times in 1× PBST for 5 min and blocked with 5% skim milk in PBS for 2 h at RT under gentle shaking. Next, serial threefold gradient dilutions of F-E2 starting at 30 nM were added and incubated for 2 h at RT under gentle shaking. The plates were subsequently washed with 1× PBST for 5 min and incubated with the secondary antibody (Rabbit Anti-Human IgG-Fc HRP; Sino Biological; 10702-T16-H-100) for 1  h at RT under gentle shaking. Afterward, the plate color was developed by adding 100 µL TMB substrate solution (Beyotime; P0209) for 5  min at RT in the dark. The reaction was terminated by adding 50 µL of 0.5 M sulfuric acid. The absorbance value was detected for each well at a wavelength of 450 nm using a Multiskan FC microplate reader (Thermo Scientific).

### Flow cytometry analyses

Vero cells were seeded in 12-well plates. After 24 h, RSV was diluted in DMEM and added to the cells for infection. After 3 h, the cells were cultured in normal medium supplemented with 2% FBS. The infected Vero cells were harvested at 60 h post-infection. Then, 4% polyformaldehyde (PFA) was slowly added to resuspend cell pellets, which were fixed for 15 min at RT. The fixed cells were centrifuged at 1,000 rpm for 5 min and washed twice with 1× PBS. After that, 5% skim milk and permeabilization buffer were used for blocking and permeabilized. Next, the cell pellets were resuspended in 1× PBS containing F-E2 antibody and incubated at RT for 1 h. Fluorescein isothiocyanate (FITC)-Goat anti-human IgG (Proteintech; SA00003-12) was added and incubated at RT for 1 h in the dark. Then, 400 µL PBS was added to the tube and mixed gently. Finally, the cell samples were detected by flow cytometry (CytoFLEX, USA), and the raw data were analyzed by using FlowJo_V10_CL. At least three independent experiments were conducted in the analysis.

### Immunohistochemistry assay

BALB/c mice were slightly anesthetized with isoflurane and infected intranasally with 1 × 10^6^ plaque-forming units per mouse of RSV A2. The mice were euthanized after 4 days. Then, the whole lung tissue was harvested and fixed in 4% PFA. The sample was dehydrated and embedded in paraffin blocks. The paraffin sections (thickness, 4 µm) were deparaffinized. The paraffin sections were deparaffinized, rehydrated, subjected to antigen retrieval, and blocked. Then, the primary antibody F-E2 was added to the slice, which was incubated at RT for 1 h. The tissue slices were washed with PBS three times and subsequently incubated with the secondary antibody (Zsbio, #PV-6000) for 20 min at RT. Finally, the color reaction was carried out by using 3,3-diaminobenzidine solution (Zsbio, #ZLI-9018) and visualized using a microscope system (Nikon, Nikon Eclipse 50i).

### Indirect immunofluorescence assay

First, Vero cells were seeded in 96-well culture plates and maintained at 37°C. When the cell density reached 70%–80%, the virus was diluted in DMEM and added to target cells for infection. After 1 h, the cells were overlaid with DMEM containing 2% FBS and incubated for 48 h. The cells were fixed overnight at 4°C. The fixed cells were washed three times with 1× PBST for 5 min each time. Then, the cells were blocked and stained with the nanobody F-E2 (10 ng/mL) at RT for 1 h, followed by three washes with 1× PBST. The samples were incubated with the secondary antibody, FITC-Goat anti-human IgG (Proteintech; SA00003-12), for 1 h at RT in the dark. Finally, the cell nuclei were labeled with DAPI, and the cell plates were imaged using the ImageXpress MicroConfocal microscope (Moleculardevices; 76177-140).

### Statistical analysis

All the statistical analyses were conducted using GraphPad software. To assess the statistical significance of the difference between two treatments, we used unpaired, two-tailed Student’s *t* tests. Statistical significances are shown as *P  <  0.05, **P  <  0.01, and ***P  <  0.001, and data are presented as the mean ± SEM.

## RESULTS

### Antigen production, immunizing camel, and building library

**Fig 1:**
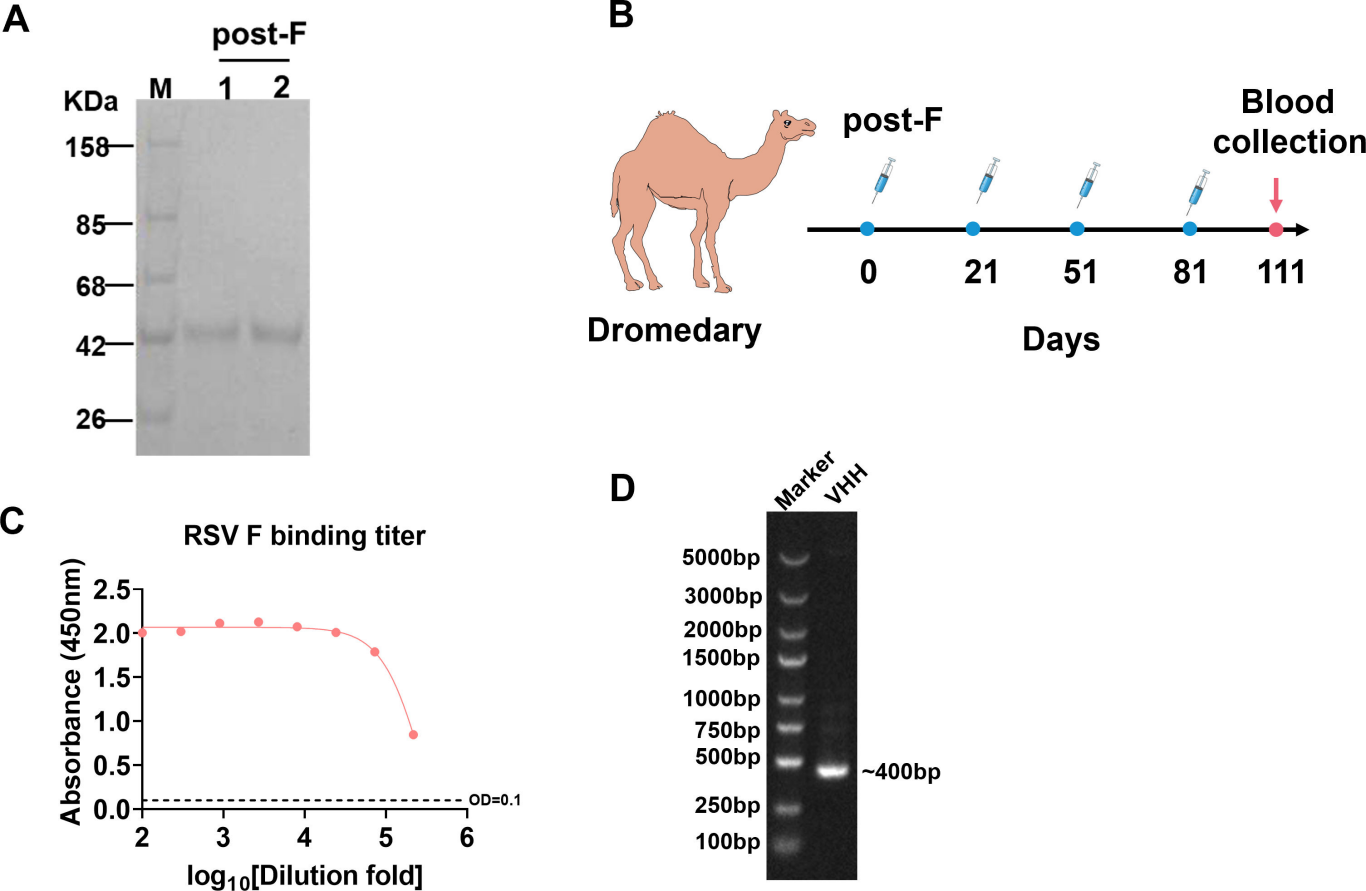
Purification of RSV F, immunization of dromedary camel, and library construction. (A) Reduced SDS-PAGE analysis of the purified RSV F (post F). (B) Schematic diagram of the immunization and blood collection schedule in dromedary camel. (C) Following immunization, serum samples were collected to assess RSV F-specific binding antibody titers by ELISA. (D) Identification of VHH sequence by PCR amplification of the nanobody.

To obtain an RSV detection antibody with favorable biological properties, the DNA sequence of the F protein labeled with His-tag was cloned into a mammalian expression vector PTT5, which was expressed by the HEK 293F cells. The antigen protein was collected from the cell supernatants by using a Ni NTA column, and the protein purity was initially determined by reduced SDS-PAGE. The result showed that the target band was single without impurities (Fig. 1A), and the highly purified RSV F protein was used to immunize the camel.

We immunized a dromedary camel four times with highly purified antigen protein at the dose of 0.5 mg/time (Fig. 1B). Next, the serum antibody titers were measured by ELISA. The studies suggested that when the OD_450_ was 0.1 and the corresponding serum dilution ratio exceeded 10,000, the immune effect was good (Fig. 1C). Blood was collected, PBMCs were isolated, and total RNA samples were extracted and reverse-transcribed into cDNA. Specific primers were used for PCR amplification to produce VHH coding regions with a size of about 400 bp, which was the gene sequence of nanobody (Fig. 1D). According to the previous description (19), the VHH fragment was cloned into the phagemid vector PR2, and the recombinant plasmid was electro-transformed into the *E. coli* TG1, and the phage library containing the nanobody fragment was generated.

### Screening, expression, and purification of nanobodies

**Fig 2:**
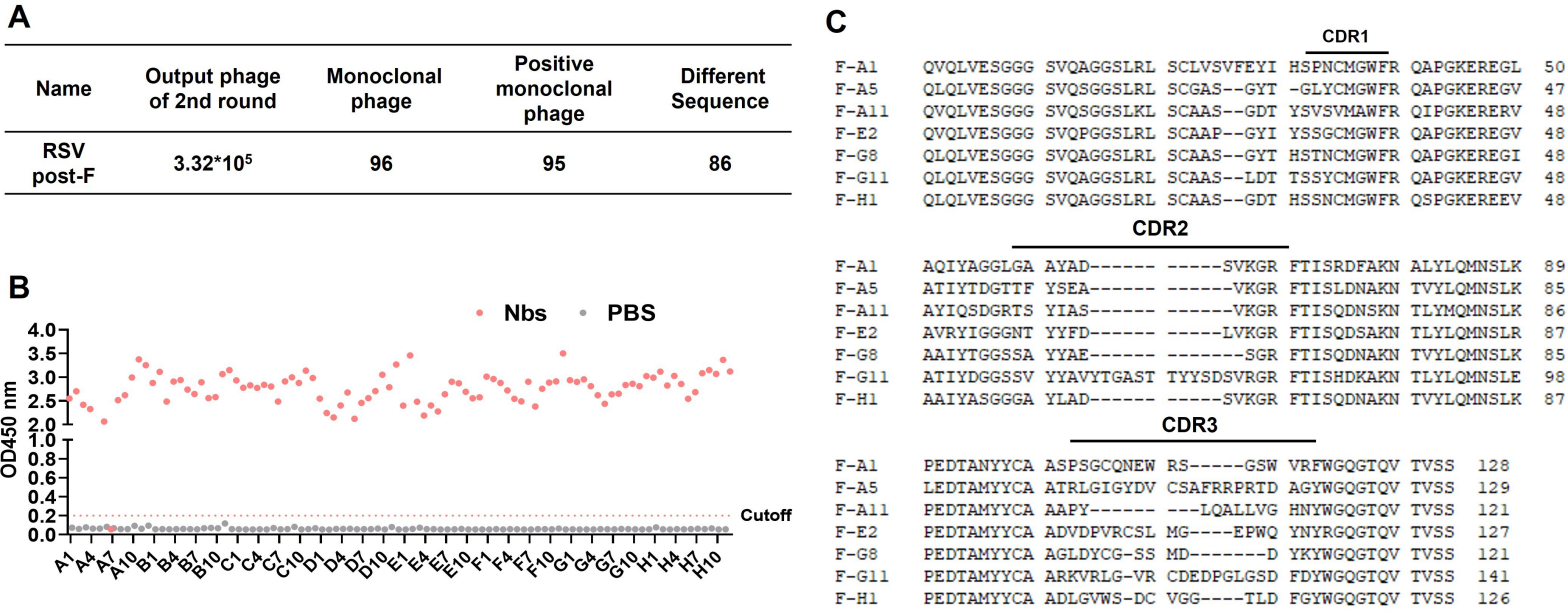
Isolation of candidate nanobodies from the phage display library of immune camel. (A) Enrichment of phages after two rounds of panning on RSV F. (B) Characterization of the binding of the isolated 96 monoclonal phages by ELISA. The cutoff value was defined as four times the optical density of control wells. (C) The amino acid sequence information of the isolated seven candidate nanobodies.

In order to obtain RSV F-specific nanobodies, we performed two rounds of panning and finally obtained a library of 3.32 × 10^5^ clones. Then, we conducted monoclonal phage ELISA, which further screened 95 positive antibodies, and 86 individuals with completely different sequences were obtained by sequencing (Fig. 2A and B). Based on the differences in the sequences and the affinities, seven candidate nanobodies were ultimately identified as targets (Fig. 2C).

**Fig 3:**
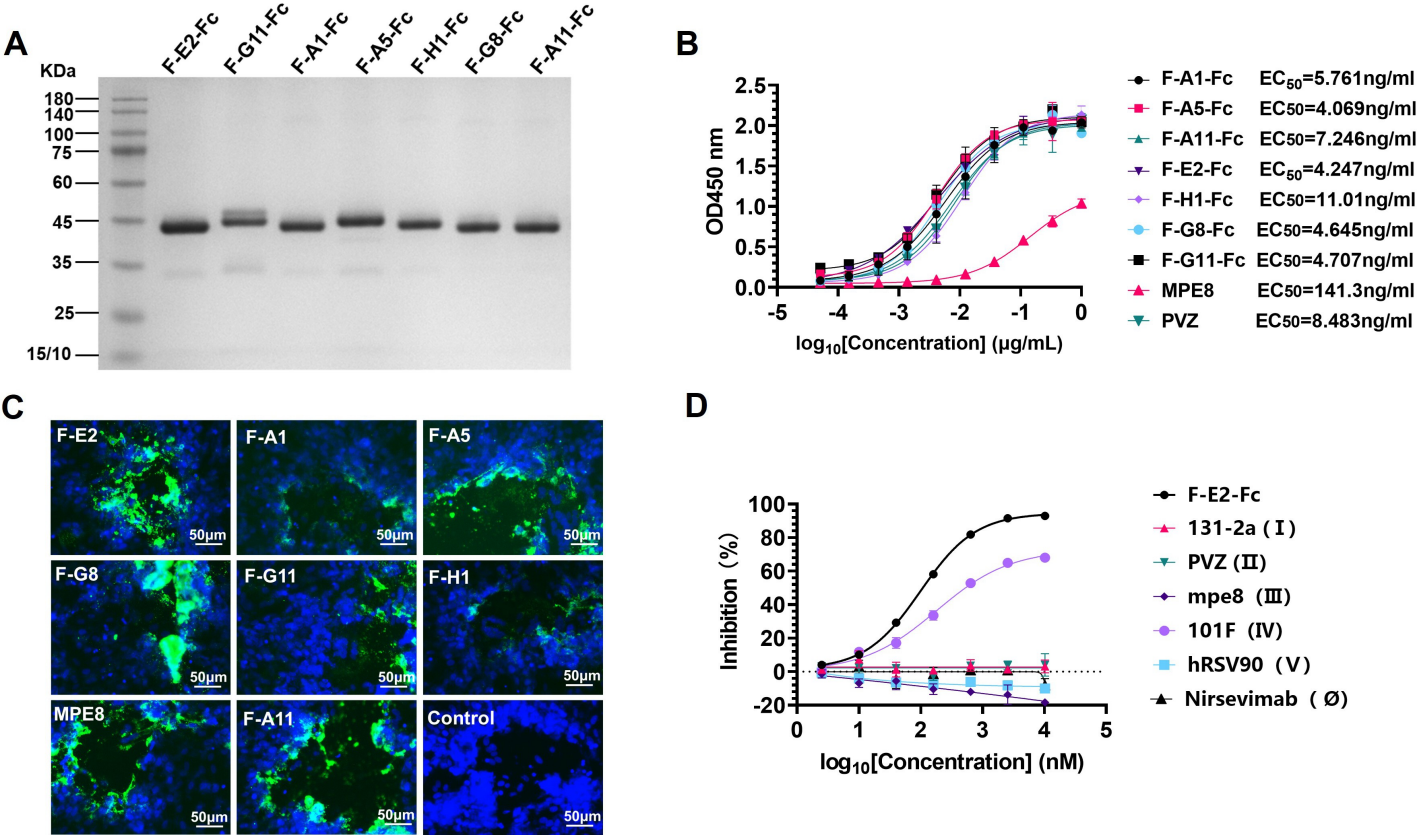
The candidate nanobodies bind to RSV F with ng affinities. (A) Reduced SDS-PAGE analysis of the seven purified candidate nanobodies. (B) Binding of the candidate antibodies to RSV F proteins. The half-maximal effective concentration (EC_50_) was calculated by GraphPad Prism 8.0 software. (C) Representative immunofluorescence images of seven candidate nanobodies. Nuclei were stained with DAPI (blue). Scale bar: 50 µm. (D) Results of competitive ELISA between F-E2 and six known epitope-specific antibodies. The values are normalized as competitive rates related to the negative control.

We further explored the biological characteristics of the candidate nanobodies. Therefore, we constructed candidate nanobodies into IgG-like molecules by fusing the C-terminus of the antibody with the cleavage site of TEV protease and human IgG1 Fc. The construct was cloned into PTT5, and homo-bivalent Nb-TEV-Fc fusion proteins were collected from the cell supernatants using the Protein A column. Finally, the protein purity was preliminarily determined by reduced SDS-PAGE. The results showed that the candidate antibodies had a single band without impurities (Fig. 3A).

### The candidate nanobodies bind to RSV F with ng affinities

To explore the binding activity of the seven candidate nanobodies, we constructed candidate nanobodies into IgG-like molecules (F-E2-Fc). We used RSV F (post F) as antigen and performed ELISA. It was found that the EC_50_ values of most nanobodies ranged from 4 to 11 ng/mL. The research indicated that the indirect immunofluorescence assay frequently employs MPE8 and palivizumab antibodies as primary antibodies (20–22). Nevertheless, the affinity of the majority of candidate nanobodies selected from the library surpasses that of MPE8 and palivizumab. In summary, the candidate antibodies have a high affinity for binding to RSV F (Fig. 3B).

To further determine whether the candidate nanobodies could bind to the native RSV F protein on the membrane, we carried out an immunofluorescence assay. The results indicated that the fluorescence signal of F-E2 was clear and could specifically recognize the native F protein (Fig. 3C). To date, RSV F has at least six different binding sites, including Ø, I, II, III, IV, and V (23). Then, we used six RSV F antibodies with known antigen-binding regions as competing antibodies as follows: 131-2a (I) (24), palivizumab (II) (25), MPE8 (III) (21), 101F (IV) (26), hRSV90 (V) (27), and MEDI-8897 (Ø) (28), respectively. F-E2 was labeled with His-tag as a blocking antibody and used for competitive ELISA. The range of the F-E2 binding site was further narrowed. The results show that F-E2 exhibits a distinct competitive relationship with 101F (IV), and F-E2 likely binds to site IV of RSV F (Fig. 3D).

### Establishment and optimization of an indirect immunofluorescence assay for determining respiratory syncytial virus titer

**Fig 4:**
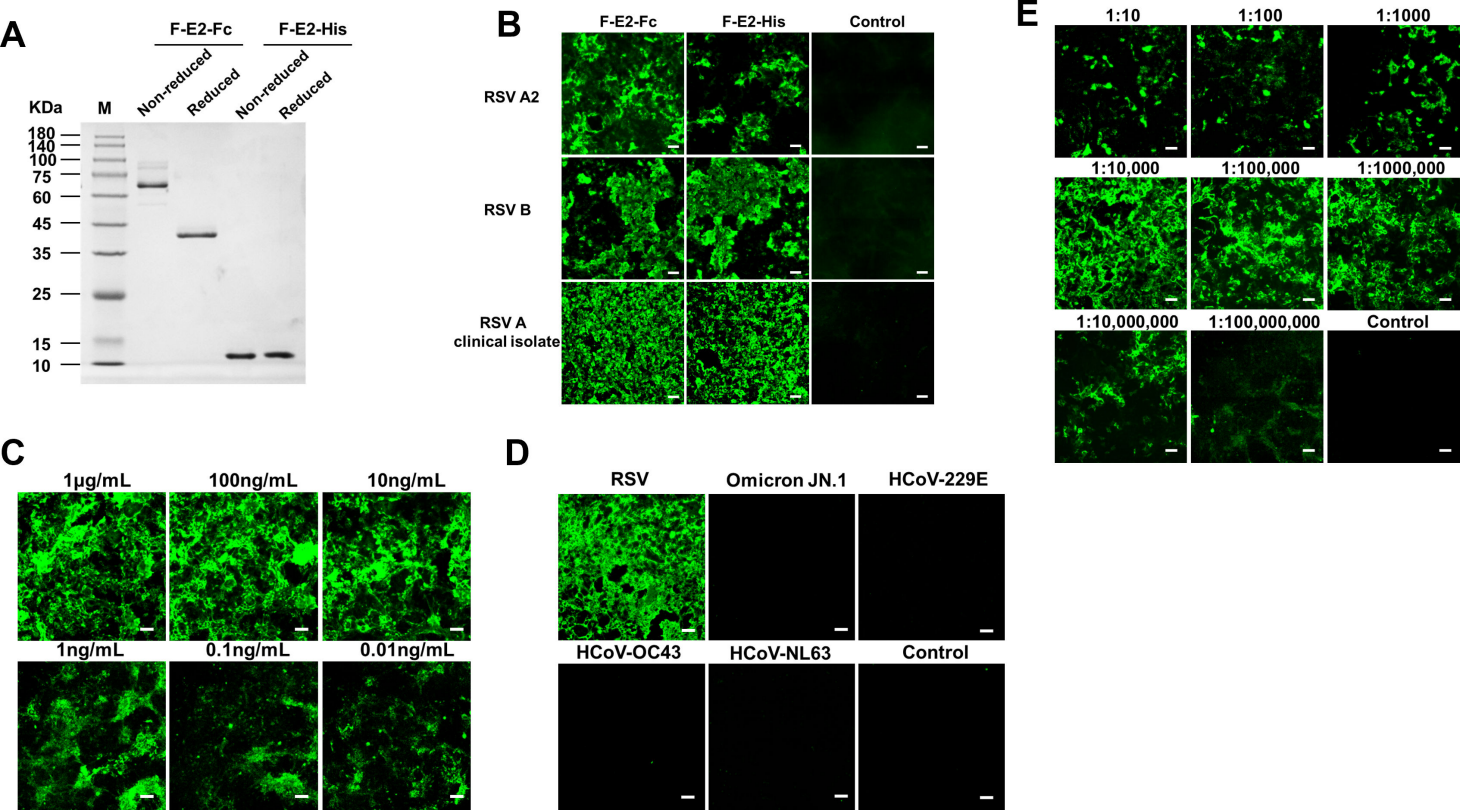
The establishment and optimization of an indirect immunofluorescence assay for determining respiratory syncytial virus titer. (A) Non-reduced and reduced SDS-PAGE analysis of the purified F-E2-Fc fusions or F-E2-His. (B) Representative immunofluorescence images of F-E2 (Fc or His tagged) for different RSV laboratory strains A2, B, and RSV A clinical isolate. Scale bar: 100 µm. (C) Indirect immunofluorescence assay of nanobody F-E2-Fc against RSV F protein at different dilutions. (D) Specific test results of indirect immunofluorescence assay. (E) Sensitivity test results of indirect immunofluorescence assay. Scale bar: 100 µm.

Considering that F-E2 specifically binds to RSV F, we further investigated the effect of different labels on the binding ability of F-E2. First, F-E2 was fused to IgG1 Fc fragments and purified from culture supernatants with protein A. F-E2 was 6×His-tagged and obtained from the flow through of Ni NTA (Fig. 4A). The results of the immunofluorescence assay showed that F-E2-Fc or F-E2-His can bind specifically to native F protein of different RSV laboratory strains A2, B, and RSV A clinical isolate (GZ08-18, GenBank accession No. KP119747) (Fig. 4B). These findings indicated that F-E2 can be a useful reagent for RSV detection.

To further investigate the detection limit and sensitivity of the F-E2 antibody, we initially diluted the nanobody F-E2 at a concentration of 1 µg/mL in gradient dilutions, followed by incubation with cells infected with RSV. The results indicate that the nanobody F-E2 retains a distinct fluorescence signal at a working solution concentration of 10 ng/mL, suggesting its suitability for immunofluorescence experiments at the nanogram level (Fig. 4C). In contrast, conventional detection antibodies, such as palivizumab, require usage at the microgram level. These findings further indicate that the F-E2 nanobody demonstrates a cost-effective and efficient profile.

In addition, RSV A2 (2 × 10^7^ TCID_50_/mL) was diluted in a 10-fold gradient with incomplete medium, and 50 µL of virus dilution solution was added to each well. Importantly, owing to the high virus concentration, which leads to cell pathological changes and full shedding, the initial three wells presented relatively low fluorescence signals. However, when the virus concentration was diluted to 10^8^ (Fig. 4E), characteristic specific green fluorescence remained detectable under a microscope, further validating the exceptional sensitivity of the nanobody F-E2.

Excellent antibodies have target specificity and sensitivity, even being able to recognize the target protein at low expression levels. However, an increasing number of studies have shown that not all antibodies have target specificity, and many antibodies also exhibit cross-reactivity with off-target proteins. Therefore, we collected different respiratory-related viruses, such as RSV, SARS-CoV-2 Omicron JN.1, HCoV-229E, HCoV-OC43, and HCoV-NL63, and added the nanobody F-E2 to cells infected with these viruses. The results showed that cells infected with OC43, 229E, and NL63 viruses did not show positive signals, whereas cells infected with RSV showed obvious specific fluorescence signals (Fig. 4D), indicating that the RSV immunofluorescence detection method based on F-E2 has excellent specificity.

### A neutralization titer detection method for RSV was established on the basis of F-E2

**Fig 5:**
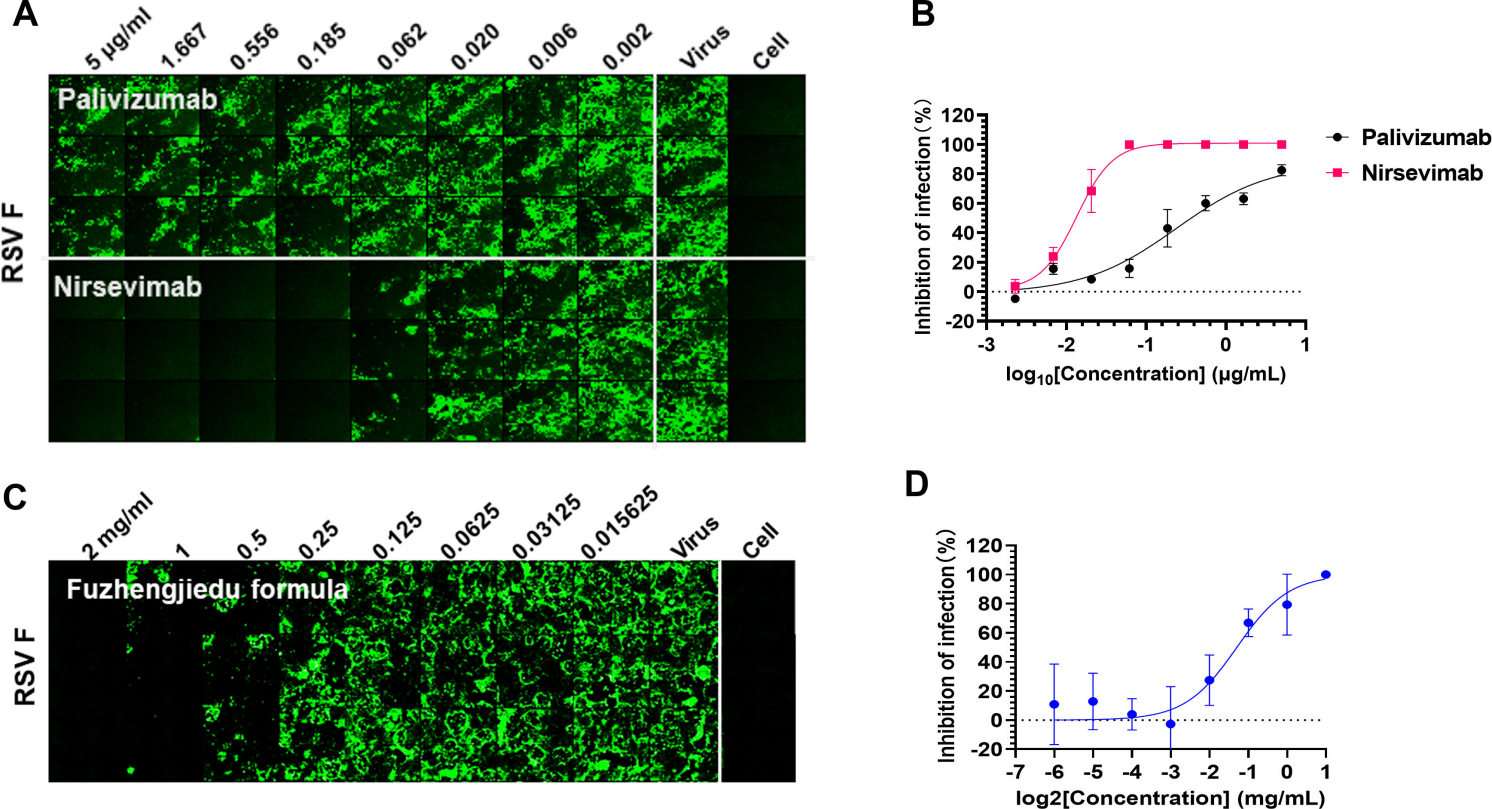
The neutralization titer detection method for RSV was established based on F-E2. (A) The neutralization assays of palivizumab and nirsevimab against RSV A2 were detected by indirect immunofluorescence assay. (B) Neutralizing curves of palivizumab and nirsevimab to RSV A2. The half maximal inhibitory concentration (IC_50_) was calculated using GraphPad Prism 8.0 software. (C and D) The neutralization assays and analysis of the Fuzheng jiedu formula against RSV A2 were detected by indirect immunofluorescence assay.

To investigate the potential application of F-E2 in detecting neutralizing titers within the RSV neutralization assay, we selected two commercially available RSV prophylactic antibodies, palivizumab and nirsevimab, which served as our positive controls. Palivizumab is a humanized monoclonal antibody exhibiting potent neutralizing activity against RSV; it is recommended for premature infants, immunocompromised children, and elderly patients with pulmonary-related conditions (29, 30). In contrast, nirsevimab is a fully human monoclonal antibody optimized from the D25 monoclonal antibody and binds to a unique epitope site Ø of the RSV F in its prefusion state. Nirsevimab exhibits a remarkably potent neutralizing effect against RSV, with its neutralizing activity against clinical isolates approximately 50 times greater than that of palivizumab (28). Subsequently, we established eight gradients, serial fourfold gradient dilutions of antibody starting at 1 µg/mL, followed by incubation with the virus prior to the addition of cultured cells. Ultimately, we assessed the RSV titer using an immunofluorescence assay. The results demonstrated that both palivizumab and nirsevimab exhibited neutralizing effects against RSV A subtype, with IC_50_ values recorded at 240.2 and 13.48 ng/mL, respectively (Fig. 5A and B).

To further validate the universality of this method, we selected an antiviral drug, the Fuzheng jiedu formula. Previous studies have shown that the Fuzheng jiedu formula has an anti-COVID-19 effect and the ability to repair lung injury (31, 32). To investigate whether the Fuzheng jiedu formula had any anti-RSV effect, we diluted the herbal formulation in graded concentrations and incubated it with RSV before adding it to the Vero cells. The results showed that the mean viral load decreased when the concentration of Fuzheng jiedu formula increased, and the Fuzheng jiedu formula significantly inhibited the replication of RSV, with IC_50_ values recorded at 0.4073 mg/mL (Fig. 5C and D). These further substantiate the applicability of F-E2 in RSV neutralization assays.

### Versatile applications of nanobody F-E2

**Fig 6:**
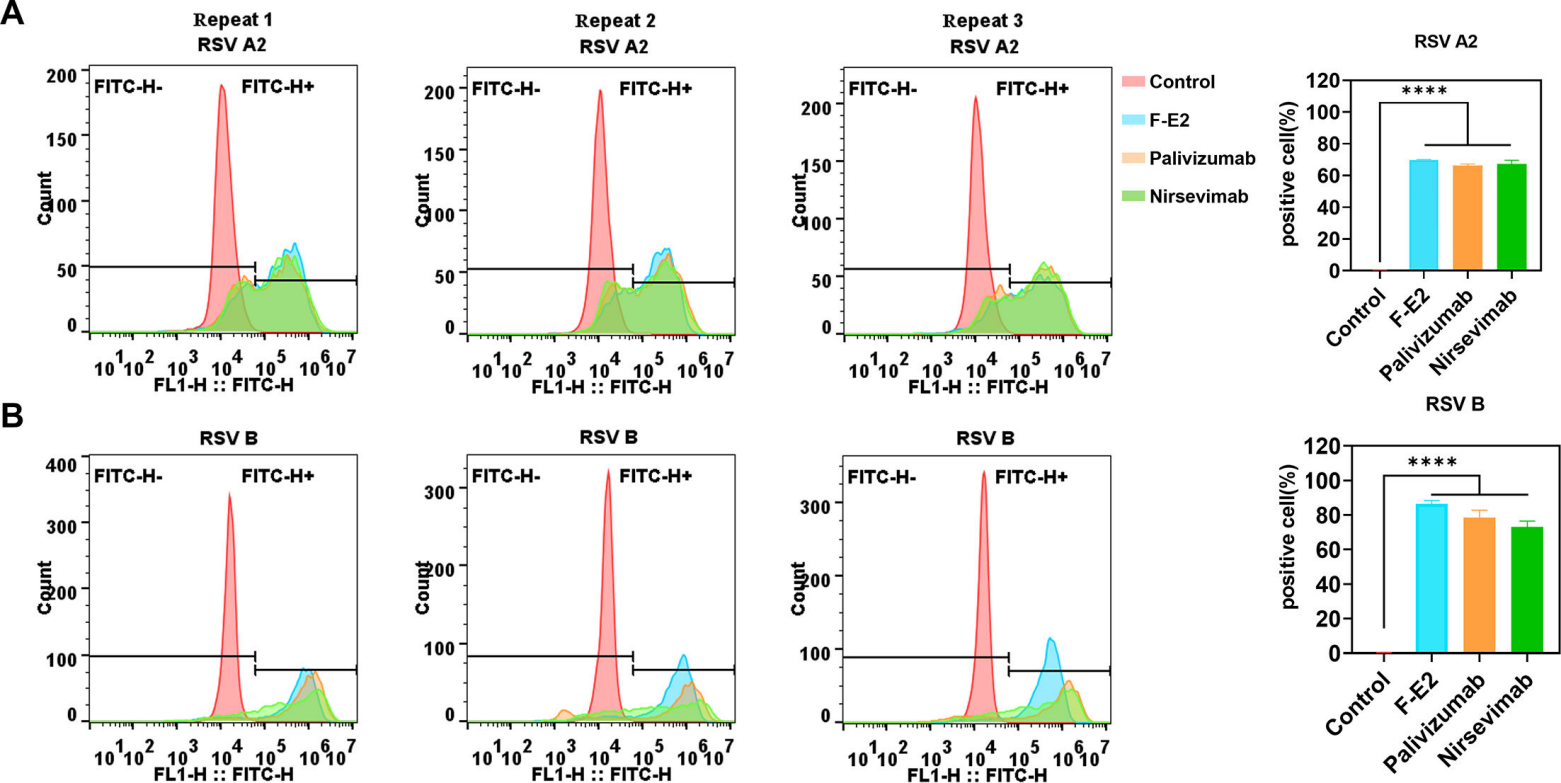
The nanobody F-E2 is applicable for the cell sorting of RSV-infected cells. (A and B) Vero cells infected with RSV A2 and B strains were sorted by flow cytometry analysis. Cells infected with RSV were incubated with F-E2, palivizumab, and nirsevimab and stained with FITC-conjugated human anti-human IgG. The proportion of FITC-positive cells among the total cells was analyzed using the FlowJo V10 software.

**Fig 7:**
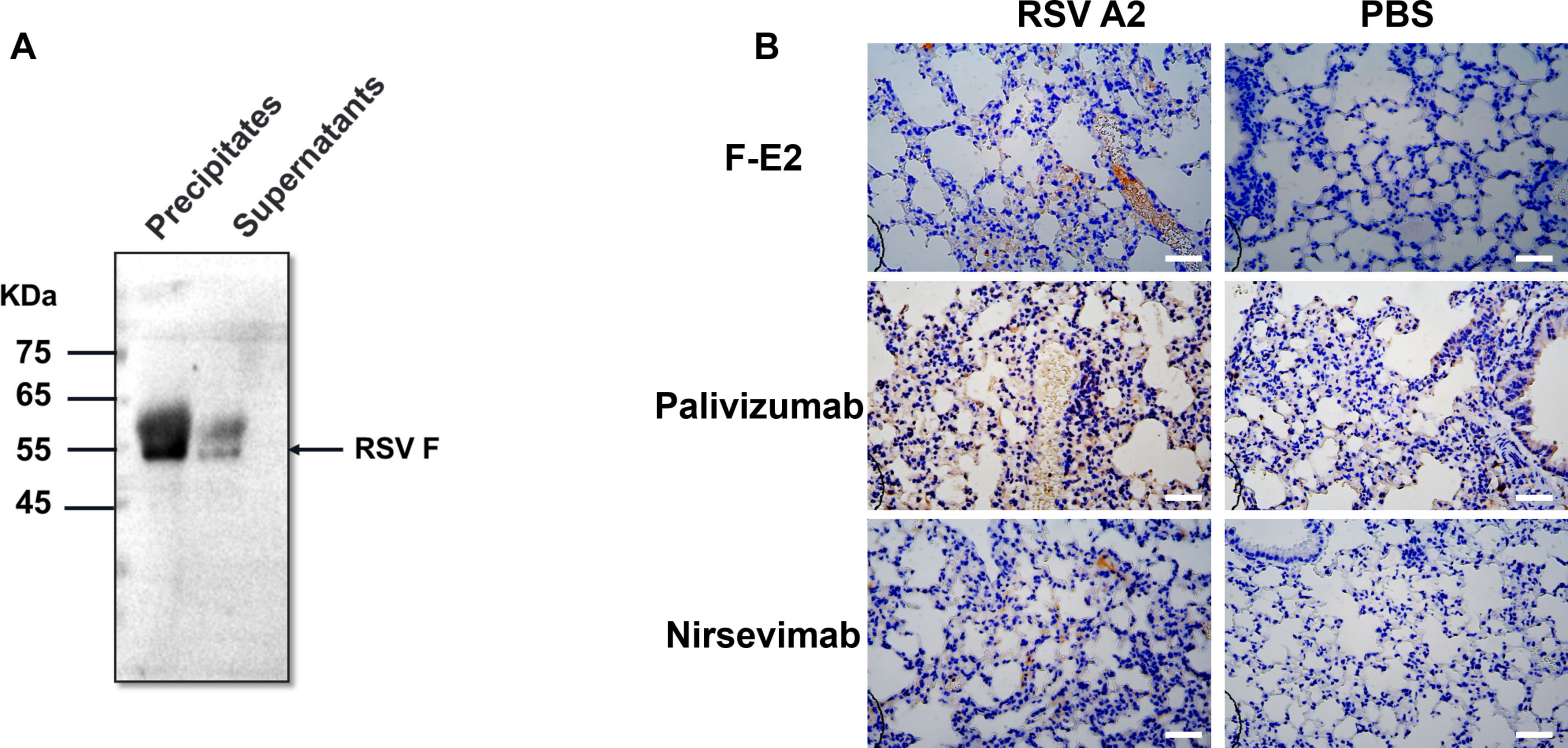
The nanobody F-E2 can be used for Western blot and IHC *in vivo*. (A) Western blot analysis of the interaction of F-E2 with RSV F. (B) Representative IHC images of F-E2, palivizumab, and nirsevimab in the lung tissue of infected mice. Scale bar: 100 µm.

Antibodies are integral to a wide array of applications in the detection and separation of proteins and small molecules, encompassing techniques such as Western blotting, ELISA, single-cell sorting, and more. To further assess the suitability of nanobody F-E2 for cell flow cytometry assay, we collected cells infected with RSV A2 and B strains and labeled them with F-E2 as the primary antibody. Moreover, we also added two positive controls to the flow cytometer, including palivizumab and nirsevimab. We found that the number of positive cells labeled with F-E2 was higher for two different RSV subtype strains, suggesting better binding activity of F-E2 than the positive control antibody (Fig. 6A and B). Additionally, F-E2 can be used in Western blot experiments. Precipitates and supernatants from RSV-infected cells were collected separately, fixed, and lysed to prepare protein extracts. The results indicate that F-E2 specifically binds to the linear epitope of the RSV F protein (Fig. 7A). More importantly, F-E2 can also be applied to IHC experiments in mouse tissues. We obtained lung tissues from mice infected with RSV A2 and subjected them to paraffin embedding and staining. The results showed a high background signal despite the positive signal of palivizumab being strong. In contrast, the specificity of F-E2 was better (Fig. 7B). In conclusion, F-E2 can be used in conjunction with a variety of RSV detection methods and is more specific and universal.

## DISCUSSION

In this study, an RSV detection nanobody F-E2 was successfully screened by constructing a camel immune library and using phage display technology. F-E2 can specifically bind to RSV F and recognize antigenic epitope IV. Subsequently, a simple, rapid, and cost-effective RSV titer detection method was established using F-E2 as an immunofluorescence primary antibody, which is conducive to the early identification of RSV infection and is an important measure for reducing RSV transmission. In addition, this method can also be used to determine the neutralization titers of candidate antibodies and provide a scientific basis for screening neutralizing antibodies and therapeutic drugs for RSV. There are various methods for measuring RSV titers. The plaque assay is a classical method in which the virus titer is determined by calculating the number of plaques formed by virus-infected cells. However, RSV plaque formation is not obvious, and it is also difficult to count them with the naked eye or microscope. When several virus particles infect a cell, it is difficult to distinguish a single plaque, which affects the accuracy of titration. In addition, the most commonly used RT-qPCR assay has the advantages of high sensitivity and specificity and can detect viral infection at an early stage; however, the cost is relatively high, the results are unintuitive, and the false-positive results are prone due to aerosols. Therefore, it is necessary to develop other methods for detecting RSV, such as indirect immunofluorescence assay, which has the characteristics of good specificity, high stability, accuracy, and intuitiveness and is widely used in pathological detection, clinical examination, and pathogen detection.

Studies have shown that the monoclonal antibody MPE8 can effectively cross-neutralize human RSV and metapneumovirus, bovine RSV, and pneumonia virus of mice (21). At present, MPE8 has been used as an antibody for indirect immunofluorescence detection of RSV in neutralization experiments (20), but there is still a lack of effective RSV detection antibodies in the domestic and foreign markets. To overcome this shortage further, we planned to screen targets with better staining effects from the nanobody library, and MPE8 was used as a positive control. The results showed that F-E2 had a low background signal, strong fluorescence signal, and obvious cytopathic effect, and the fluorescence intensity exhibits a positive correlation with the increasing viral load. The staining effect of other nanobodies was poor, with non-specific fluorescence, low fluorescence intensity, and unclear cell boundaries. The result showed that F-E2 has a higher ability to bind RSV F than other antibodies, with EC_50_ values around 0.017 nM, which may be the reason why it is a candidate for fluorescence detection of antibodies; however, the specific molecular mechanism needs to be further explored through protein structure analysis.

VHH retains intact antigen binding ability and has a molecular weight of only 12–15 KDa, which is 1/10th of the traditional antibodies (33). F-E2 was fused to IgG1 Fc fragments, and we computed the molecular weight of F-E2-Fc to be about 43 KDa by software “expasy.” Although the molecular weight of the F-E2 increases after combining with a human Fc, it is still much smaller than that of traditional antibodies. More importantly, a homo-bivalent nanobody-TEV-Fc fusion protein has many advantages as follows: (i) the fusion of the nanobody with IgG1 Fc resulted in homodimers with greatly improved binding affinities (20). (ii) The experimental cost is low, and the range of applications is wide. Secondary antibodies against IgG1 Fc, such as anti-IgG1 Fc-HRP antibody or fluorescein isothiocyanate-goat anti-human IgG, are cost-effective. (iii) A final point to consider is the addition of IgG1 Fc, which can increase the half-life of the antibody and can also be used as a marker in later live animal experiments (34, 35).

In summary, F-E2 as a fluorescent detection nanobody has the characteristics of strong fluorescence intensity, low background, and high expression level. Compared with the control monoclonal antibody MPE8, the fluorescence signal is clear, the dosage is less, and it is economical and effective. It has broad application prospects and important economic and social significance in the development of RSV infection detection reagents, targeted drug delivery, and neutralizing antibody therapy.

## AFTER EPUB

[This article was published on 25 March 2025 with an error in Figure 4. The Figure 4 was corrected in the current version, posted on 6 June 2025.]
